# Double-edged sword of FeS oxidation in estuarine and coastal sediments: accelerating elemental cycling yet enhancing antibiotic resistance enrichment

**DOI:** 10.1128/aem.01525-26

**Published:** 2026-09-30

**Authors:** Wei Qiu, Xiufeng Tang, Boshuang Wu, Haoran Huang, Xiangyang Zhao, Jun Yuan, Xiaofei Li

**Affiliations:** 1 Jiangsu Key Laboratory of Organic-based Fertilizer Creation and Soil Health Manipulation, Educational Ministry Engineering Center of Resource-Saving Fertilizers, Nanjing Agricultural University 70578, Nanjing, China; 2 State Key Laboratory of Estuarine and Coastal Research, East China Normal University 540512, Shanghai, China; University of Delaware, Lewes, Delaware, USA

**Keywords:** ferrous sulfide, ARGs, CBB cycle, nitrogen fixation, estuarine sediments

## Abstract

Biogeochemical cycles exert significant influences on the fate and behavior of antibiotics in estuarine and coastal environments; however, ferrous sulfide (FeS)-mediated enrichment and dissemination of antibiotic resistance genes (ARGs) remain poorly understood. Here, we conducted microcosm experiments and field investigations to examine the relationships of FeS amendment, microbial communities, functional potentials in relation to elemental cycling, and ARG abundance. The positive effects of FeS on ARG abundance were aerobic dependent, which were accompanied by a significant enrichment of Proteobacteria, as the crucial hosts of ARGs. Proteobacteria were associated with systematic metabolic pathways for carbon fixation (Calvin-Benson-Bassham [CBB] cycle), N_2_ fixation, and FeS oxidation. In addition, field samples from China estuarine and coastal wetlands further showed that CBB cycle, N_2_ fixation, and sulfur oxidation pathways were significantly correlated with the relative abundance of ARGs. These findings collectively indicate that FeS oxidation facilitates enrichment and dissemination of ARGs through selective enrichment of Proteobacteria and consequent enhancement of metabolic synergies between sulfur, iron, carbon, and nitrogen cycles. However, FeS-mediated oxidation may also degrade microbial DNA and suppress ARG proliferation, indicating that the net outcome of ARG enrichment depends on oxygen conditions and microbial community filtering. The cascade processes of environmental regulation-community restructuring-metabolic synergy are of great significance to enhance ARG enrichment and dissemination. Therefore, redox fluctuations can enhance elemental cycling and further promote significant enrichment and dissemination of ARGs. These findings highlight the importance of redox dynamics in ARG monitoring and risk assessment in estuarine and coastal wetlands.

**IMPORTANCE:**

Estuarine and coastal wetlands are important ecosystems for antibiotic resistance gene (ARG) enrichments. This study investigated FeS oxidation-mediated biogeochemical cycling and ARG dissemination. FeS oxidation increased enrichment and dissemination of the ARGs in estuarine and coastal sediments. The FeS oxidation reshaped microbial community structure and selectively enriched the critical Proteobacteria, which played the crucial hosts of the ARGs. The enrichment and dissemination of ARGs in the presence of FeS oxidation were due mainly to the enhanced metabolic coupling of sulfur oxidation, nitrogen fixation, and carbon fixation. CBB cycle, N_2_ fixation, and sulfur oxidation pathways were also significantly correlated with ARG abundance along the latitudinal gradient. Therefore, enhanced elemental metabolic pathways under FeS oxidation exacerbated enrichment and dissemination of ARGs, highlighting the coupling mechanisms of biogeochemical cycle and ARGs in estuarine and coastal wetlands.

## INTRODUCTION

Human activities have increased antibiotic loadings in the Earth environment, which are accompanied by a significant enrichment of antibiotic resistance genes (ARGs) and have attracted considerable attention in environments (1–4). Estuarine and coastal environments are the critical ecological niches connecting terrestrial and marine ecosystems, which have greatly suffered from antibiotic pollution due to their widespread transformation and dissemination through water discharge (2). In addition, fishery activities use antibiotics to prevent diseases and further increase commercial production, making large accumulations of antibiotics and widespread dissemination in estuarine and coastal environments. Consequently, estuarine and coastal sediments can not only accumulate substantial antibiotics, and also are assumed hotspot settings for the enrichment and dissemination of ARGs due to dynamic redox conditions (5–8). It is well acknowledged that redox fluctuations can enhance biogeochemical cycling; however, how the strengthened processes mediate the ARG enrichment and dissemination remains to be further clarified (9, 10).

Ferrous sulfide (FeS) is an iron mineral in anoxic sediments and has a profound effect on the migration and transformation of pollutants (11–13). The tidal fluctuations can shape oxygen concentration gradient and cause reducing conditions in estuarine and coastal ecosystems, triggering abiotic and biotic FeS oxidation (14). These processes not only link the interactions between electron transfer and mineral phase transformation but also yield reactive oxygen species (ROS, such as ·OH), as well as changes in local microenvironmental pH and redox potential (Eh) (13, 15). Such changes constitute intense environmental selection pressures, directly reshaping the composition and function of microbial communities and exerting interactive effects on ARGs (16, 17). However, how the cascading effects of environmental conditions, biogeochemical processes, and microbial communities influenced by FeS oxidation mediate the enrichment and dissemination of ARGs remains poorly understood.

Microbial communities are the critical medium for ARGs, in which the composition and metabolic activity greatly determine the enrichment and dissemination of ARGs (18, 19). Previous studies have shown that specific microbial taxa, such as Proteobacteria and *Bacteroidota*, are the common hosts for various ARGs (20–22). Estuarine and coastal sediments are enriched in organic matter and characterized by dynamically changing redox conditions, which are favorable to the FeS oxidation and further provide a critical energy source for microbial metabolisms (13). This energy-harnessing mechanism can directly drive metabolic processes such as carbon fixation and N_2_ fixation, which are substantially responsible for biomass synthesis and microbial proliferation (23, 24). It should be noted that carbon fixation and nitrogen metabolic pathways are closely associated with ARG abundance (25), representing a potential coupling between substance metabolism and antibiotic resistance. Given that FeS oxidation is highly strengthened under tidal forces, we hypothesize that the enhanced coupling of carbon, nitrogen, and sulfur cycling processes can facilitate microbial community restructuring and metabolic reprogramming and further increase enrichment and dissemination of ARGs in estuarine and coastal environments. Previous studies on iron-sulfur mineral oxidation have mainly focused on heavy metal-associated co-selection and ROS-mediated damage to microbial cells. However, how FeS oxidation promotes ARG enrichment through the coupling of carbon, nitrogen, and sulfur metabolic pathways and the consequent enrichment of ARG-hosting taxa remain poorly understood.

In light of sparse knowledge of the enrichment and dissemination mechanisms of ARGs in estuarine and coastal sediments, we investigated the effects of FeS on ARGs under aerobic and anaerobic conditions through microcosm experiments. The main objectives of this study are (i) to clarify the enrichment of ARGs in the presence of FeS under redox fluctuations, (ii) to reveal the successful patterns of microbial communities in the presence of FeS oxidation, and (iii) to examine correlations between microbial metabolisms of carbon fixation, nitrogen fixation, sulfur, and abundance of ARGs in estuarine and coastal sediments. This study can provide insights into crucial constraints on the enrichment and dissemination of ARGs in estuarine and coastal environments.

## MATERIALS AND METHODS

### Study area and sample collections

Yangtze estuary is one of the largest estuaries in the world and is located on the East China coast (Fig. S1). This estuary is situated with a subtropical monsoon climate, with a mean annual temperature of 15.3°C and a mean annual precipitation of 1,100 mm. The Yangtze River has a length of 6,300 km and encompasses a watershed area of 180 × 10^4^ km^2^. Yangtze estuary is characterized by a mixed river-tide estuary, due to the regular semi-daily fluctuations, with two high tides and two low tides every 6 h. Redox fluctuations are thus potential drivers of anaerobic and aerobic interface formations and biogeochemical cycling in this estuary. Chongming Dongtan Wetland (31°25'−31°38' N, 121°50'−122°05' E) is the largest and most well-developed tidal mudflat wetland in the Yangtze estuary. Sediments were collected in Dongtan wetland in June 2023. Surface sediment samples (0–10 cm) were collected from the low intertidal setting of Chongming Dongtan wetland (Fig. S1). Sediments were sampled in three replicates. All samples were stored at 4°C and transported to the laboratory within 2–4 h. After returning to the laboratory, sediments were kept at 4°C for conducting experimental incubation and determining physicochemical properties.

### Experimental incubation

Surface sediments (0–5 cm) from the low tidal flat of Chongming Dongtan wetland were selected for the amendment incubation experiment because this layer represents the critical transition zone between anaerobic and aerobic conditions in estuarine sediments. Briefly, 300 g of fresh sediment was placed into a 500 mL brown glass jar with a septa-capped lid and mixed with overlying water to maintain a water content of 40%. Four treatments were established: (i) anaerobic + FeS, (ii) anaerobic, (iii) aerobic + FeS, and (iv) aerobic. The FeS powder (Macklin, product no. F809695) was homogeneously mixed with the sediments in an anaerobic glove box to a final concentration of 7 mg^−1^ sediment (26). Anaerobic conditions were generated by bubbling helium gas through the headspace for 30 min, followed by immediate sealing of the jars with caps. Aerobic treatments were maintained under ambient air conditions. All incubation jars were placed in a 25°C dark incubator and incubated for 91 days. During incubation, deionized water was replenished in the aerobic treatments to compensate for evaporative loss and maintain the target moisture content. After incubation, sediments were collected for microbial analysis and physicochemical property determination (Fig. S2).

### Determination of sediment properties

Water content was determined after drying at 105°C. Sediment pH was measured using a Mettler-Toledo pH meter with a soil-to-water ratio of 1:2.5 (weight/volume). Sediment was extracted with 2 mol L^–1^ KCl solution and shaken at 150 rpm for 30 min and then was centrifuged at 3000 rpm for 5 min. The concentrations of NO_2_⁻, NO_3_⁻, and NH_4_^+^ were determined using a continuous flow analyzer (SAN plus, Skalar Analytical B.V., Breda, Netherlands). Sediments were dried and ground and then passed through a 200-mesh nylon sieve. The screened sediments were acidified with 1 mol L⁻¹ HCl to measure total organic carbon content (TOC). Dissolved organic carbon (DOC) was extracted by deionized water at a ratio of 1:5 (weight/volume). The DOC and TOC were determined using a total organic carbon analyzer (SSM-5000A, Shimadzu, Japan). Easily oxidized organic carbon (EOC) was measured by potassium dichromate oxidation method (27). Microbial biomass carbon (MBC) and nitrogen (MBN) were determined by chloroform fumigation method (28, 29). Sulfate in sediments was extracted with deionized water and determined by ion chromatography (Dionex ICS-2000, America). Sulfide was measured by methylene blue spectrophotometry method (30). Fe^2+^ and Fe^3+^ concentrations in sediments were measured using a 0.1% o-phenanthroline colorimetric method (31). Sediment particle size was measured using a laser particle size analyzer (2000, Mastersizer, UK).

### Gene catalog construction and ARG/MGE functional annotation

In this study, we used CD-HIT to de-replicate the gene set for identifying and quantitatively analyzing antibiotic resistance genes and further constructed a non-redundant gene catalog to ensure the accuracy of subsequent analyses. We used Diamond to align non-redundant gene sequences with the Comprehensive Antibiotic Resistance Database (CARD, v2.0.1) and the Mobile Element Database (mobileOG) (32), making the similarity threshold of 60% and the significance threshold of e-value <1 × 10^−10^ to strictly ensure the reliability of annotation results. Non-redundant gene sequences were aligned with the CARD using Diamond. After identifying the ARGs and mobile genetic elements (MGEs) based on the alignment results, we mapped the raw sequencing reads to the non-redundant gene set using the Salmon. The abundance of each gene was normalized by transcripts per million (TPM) to eliminate the effects of sequencing depth and gene length, thereby obtaining reliable gene abundance data (33). Finally, the relative abundance of various categories of ARGs and MGEs was calculated based on TPM values. Additionally, all gene sequences were aligned with the Kyoto Encyclopedia of Genes and Genomes (KEGG) database to explore their biological functional context and to obtain pathway annotation information.

### DNA extraction, metagenomic sequencing, assembly, and genome binning

Total DNA in sediments was extracted using the DNeasy Power Soil Kit. DNA quality and concentration were detected using the NanoDrop-2000 spectrophotometer. Subsequently, DNA was subjected to paired-end sequencing on the Illumina NovaSeq platform using NovaSeq Reagent Kits. Data quality control was performed with Trimmomatic, metagenomic sequences were assembled with MEGAHIT, open reading frames were predicted with MetaGene, and a non-redundant gene catalog was constructed with CD-HIT-EST. Representative sequences were selected by comparing with the NCBI NR database.

We processed the assembly results using an integrated binning strategy (34). Specifically, binning was first performed separately using MetaBAT2 (35), MaxBin2 (36), and SemiBin2. Subsequently, their results were integrated using DAS Tool (37) to obtain a non-redundant set of optimal genomes. Dereplication clustering of these genomes was carried out with dRep to remove highly similar strains. Open reading frames were predicted using Prokka, and functional annotation was performed on the dereplicated genomes. The completeness and contamination of all genomes were evaluated using CheckM2 (38). We further performed species taxonomic annotation of the quality-qualified genomes using GTDB-Tk based on the latest version of the genome classification database (39). Finally, quality-filtered sequencing reads were mapped back to the metagenome-assembled genomes (MAGs) with CoverM to calculate their relative abundance in each sample. In addition, this study reanalyzed a publicly available metagenomic data set (SRP341954/PRJNA771937) comprising 16 sampling sites in estuarine and coastal wetlands. This data set was originally reported by Zheng et al. (40). We used it for environmental validation in the field, focusing on ARG abundance, Proteobacteria abundance, and their associations with key metabolic pathways.

### Statistical analysis

Statistical analyses were performed using SPSS v23.1 and R v4.2.3. One-way analysis of variance (ANOVA) was used to test differences among treatments in sediment physicochemical properties, antibiotic abundance, relative abundances of ARGs and MGEs, ARG-MGE co-located gene clusters (AMCCs), and Proteobacteria abundance, followed by Tukey’s honestly significant difference (HSD) post hoc test for pairwise comparisons. To control for multiple testing in correlation analyses, *P* values were adjusted using the Benjamini-Hochberg false discovery rate method. For the SparCC-based co-occurrence network, only significant correlations with |*r*| > 0.6 and *P* < 0.05 were retained for network construction. Microbial community analyses were conducted in R v4.2.3. Alpha diversity indices were calculated using the vegan package, and beta diversity was assessed based on Bray-Curtis dissimilarity. Simple linear regression was used to examine the associations between key metabolic pathways and ARGs (*P* < 0.05). Sankey diagrams were generated to visualize links between ARGs and their potential microbial hosts. Mantel tests were performed to evaluate correlations between environmental factors and ARGs or MAGs. MAGs were retained only when they met the quality criteria of completeness >50% and contamination <10%, as estimated by CheckM2. Structural equation modeling was further used to assess the direct and indirect effects of environmental factors on ARGs and key ARG-carrying MAGs, and model fit was evaluated using standard indices, including *χ*²/df < 5, comparative fit index (CFI) ≥ 0.90, Tucker-Lewis index (TLI) ≥ 0.90, root-mean-square error of approximation (RMSEA) ≤ 0.08, and standardized root-mean-square residual (SRMR) ≤ 0.08.

## RESULTS

### Changes of ARGs and microbial communities

**Fig 1:**
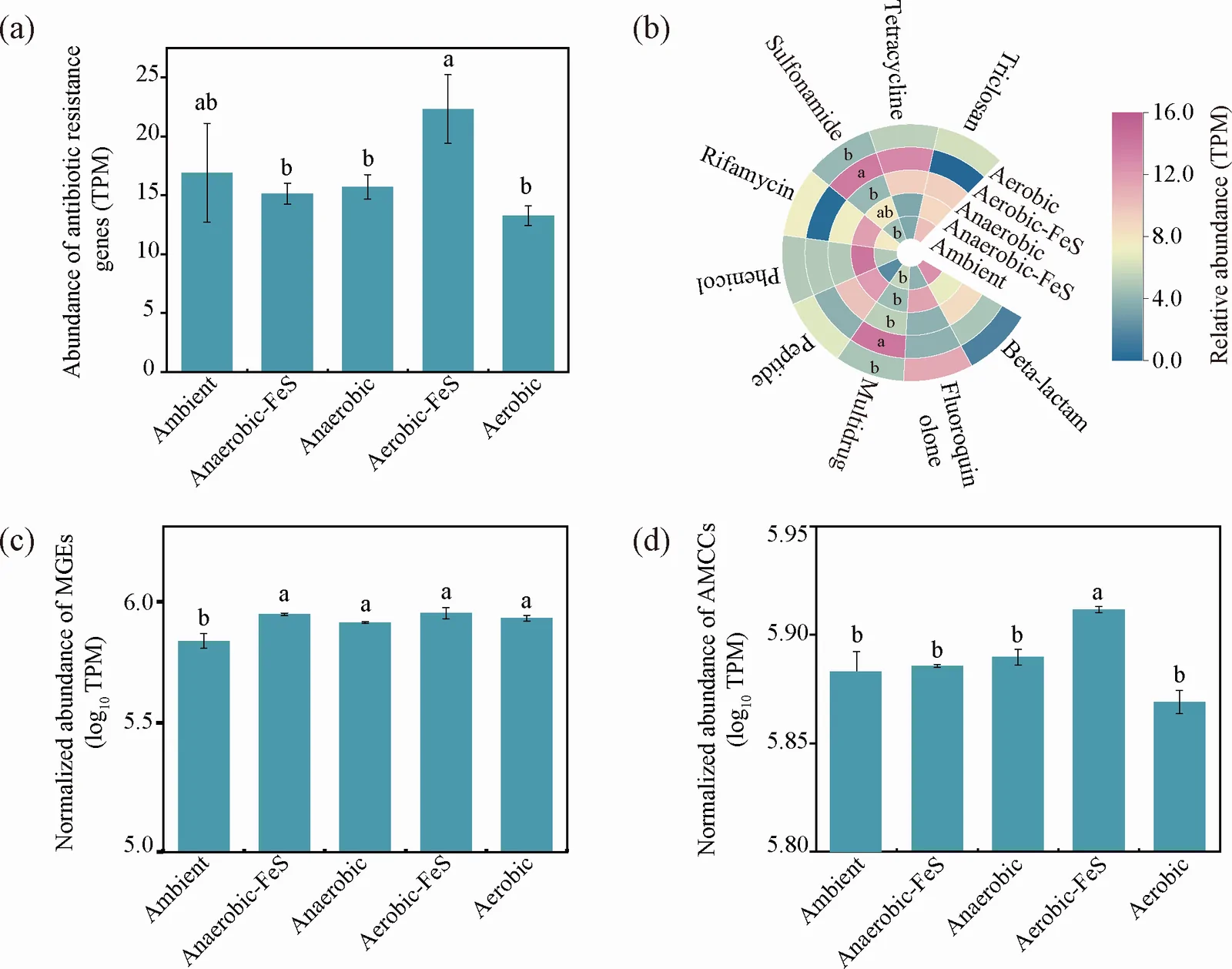
Effects of FeS on antibiotic resistance genes (ARGs). (**a**) Effects of FeS addition on the total abundance of ARGs under aerobic and anaerobic conditions. Different letters above the bars indicate significant differences between treatments (Tukey’s HSD test, *P* < 0.05). (**b**) Effects of FeS addition on the abundance of different classes of ARGs under aerobic and anaerobic conditions. Different letters indicate significant differences between treatments based on the Tukey–Kramer HSD test following ANOVA (*P* < 0.05). (**c**) Effects of FeS addition on the abundance of mobile genetic elements (MGEs) under aerobic and anaerobic conditions. Different letters above the bars indicate significant differences between treatments (Tukey’s HSD test, *P* < 0.05). (**d**) Effects of FeS addition on the abundance of ARG–MGE co-occurrence clusters (AMCCs) formed by ARGs and MGEs under aerobic and anaerobic conditions. Different letters above the bars indicate significant differences between treatments (Tukey’s HSD test, *P* < 0.05). Each treatment had three biological replicates (*n* = 3).

**Fig 2:**
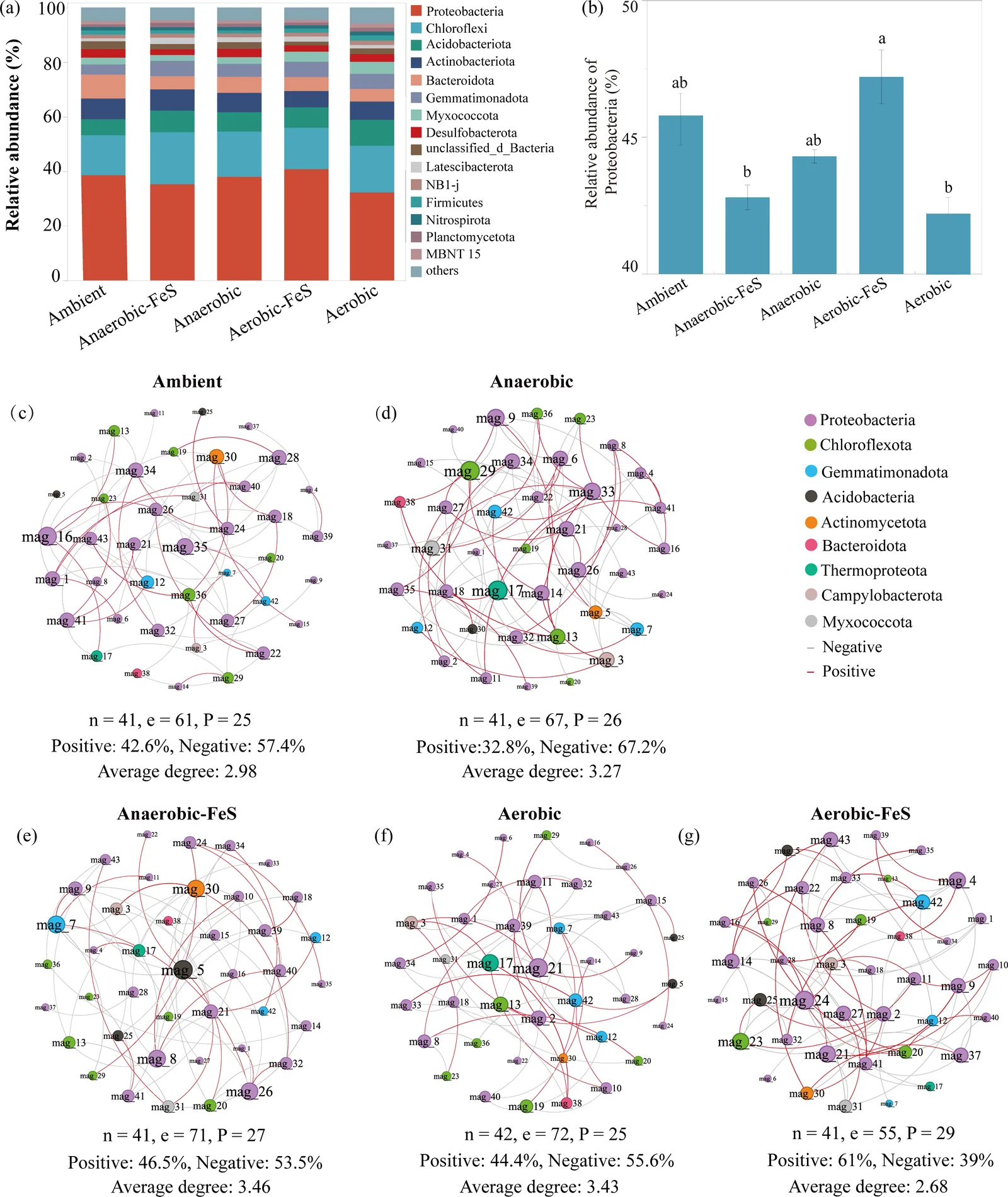
Bacterial community changes under FeS addition. (**a**) Relative abundance (%) of the major bacterial phyla in different treatment groups. (**b**) Effects of FeS addition on the relative abundance of Proteobacteria under aerobic and anaerobic conditions. Different letters above the bars indicate significant differences between treatments (Tukey’s HSD test, *P* < 0.05). Each treatment had three biological replicates (*n* = 3). (**c–g**) MAGs-based species co-occurrence networks. Nodes represent individual MAGs, and edges indicate SparCC correlations (|*r*| > 0.6). Node size is proportional to the number of connections (degree), and node colors reflect taxonomic affiliation. The letter P stands for Proteobacteria.

FeS addition significantly increased ARG abundance, as well as sulfonamide and multidrug resistance genes under aerobic conditions (Fig. 1a and b). FeS did not significantly affect the abundance of MGEs under anaerobic nor aerobic conditions (Fig. 1c). The gene clusters formed by ARGs and MGEs (AMCCs) showed that FeS significantly increased AMCCs abundance under aerobic conditions (Fig. 1d). Although the total abundance of MGEs did not change significantly under the Aerobic-FeS treatment, the abundance of AMCCs increased markedly. This indicates that FeS oxidation did not increase the overall MGEs pool, but instead strengthened the physical linkage between ARGs and MGEs at the genomic level, thereby potentially enhancing horizontal gene transfer. Significant separation in microbial structures was observed between the groups, and FeS addition reduced the microbial variation (Fig. S3a). FeS addition under aerobic conditions significantly reduced ACE, Chao, and Shannon indices (Fig. S3b). Proteobacteria, *Chloroflexi*, *Acidobacteria*, *Actinobacteria*, and *Bacteroidetes* were the dominant phyla in these groups (Fig. 2a). Relative abundance of Proteobacteria was significantly increased in the presence of FeS under aerobic conditions (Fig. 2b). The detected ARGs (22/28) were primarily derived from Proteobacteria (Fig. S3c). Despite the overall reduced complexity in the FeS + Aerobic-FeS group, this group showed the highest proportion of Proteobacteria (29/41) and the highest positive correlations among communities (61%; Fig. 2c through g). The reduced network complexity suggests strong environmental filtering under the Aerobic-FeS treatment. Meanwhile, the increased proportion of positive correlations may reflect enhanced metabolic complementarity and greater niche overlap among community members.

### MAGs and elemental metabolic potential

**Fig 3:**
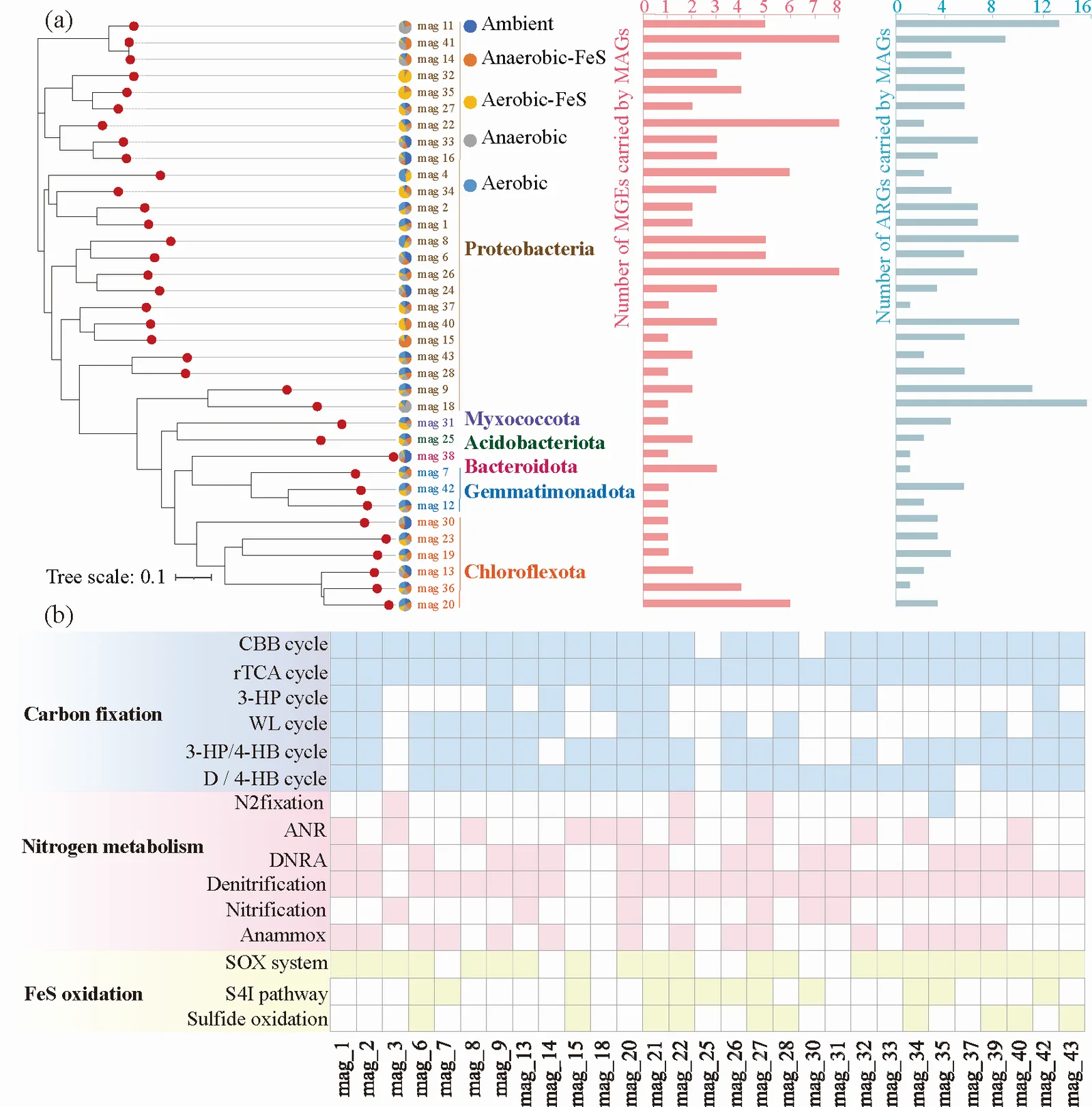
MAGs enrichment and their metabolic potentials for carbon, nitrogen, iron, and sulfur (**a**) Phylogenetic analysis of MAGs carrying both ARGs and MGEs. MAGs were color-coded at the phylum level. The outer ring (pie chart) displays the relative abundance distribution of these MAGs across different treatment groups. The bar graph indicates the numbers of MGEs (red bars) and ARGs (blue bars) contained within these MAGs. (**b**) MAGs with concurrent metabolic potential for carbon fixation, nitrogen metabolism, and iron-sulfur oxidation were identified. Metabolic functions are color-coded in blue (carbon fixation), pink (nitrogen metabolism), and yellow (iron-sulfur oxidation), with solid squares indicating that the corresponding pathway was annotated in the MAGs.

MAGs were affiliated with multiple bacterial phyla, including *Chloroflexota* (6), *Gemmatimonadota* (3), *Myxococcota* (1), *Acidobacteriota* (1), *Bacteroidota* (1), and Proteobacteria (24) (Fig. 3a). Notably, several MAGs (mag32, mag35, mag22, mag27, mag34, and mag37) affiliated with Proteobacteria were highly enriched in the presence of FeS under aerobic conditions (Fig. 3a). The functional annotation analysis showed that metabolic pathways were associated with carbon fixation, nitrogen metabolism, and FeS oxidation carried out by these MAGs. Except for mag14 and mag18, all other Proteobacteria-affiliated MAGs possessed the complete metabolic potential for carbon fixation, nitrogen metabolism, and FeS oxidation (Fig. 3b). Additionally, mag7 and mag42 affiliated with *Gemmatimonadota*, along with mag13, mag20, and mag30 affiliated with *Chloroflexota,* exhibited complete metabolic capabilities for carbon fixation, nitrogen cycling, and FeS oxidation.

### Metabolic pathways of ARGs enrichment

**Fig 4:**
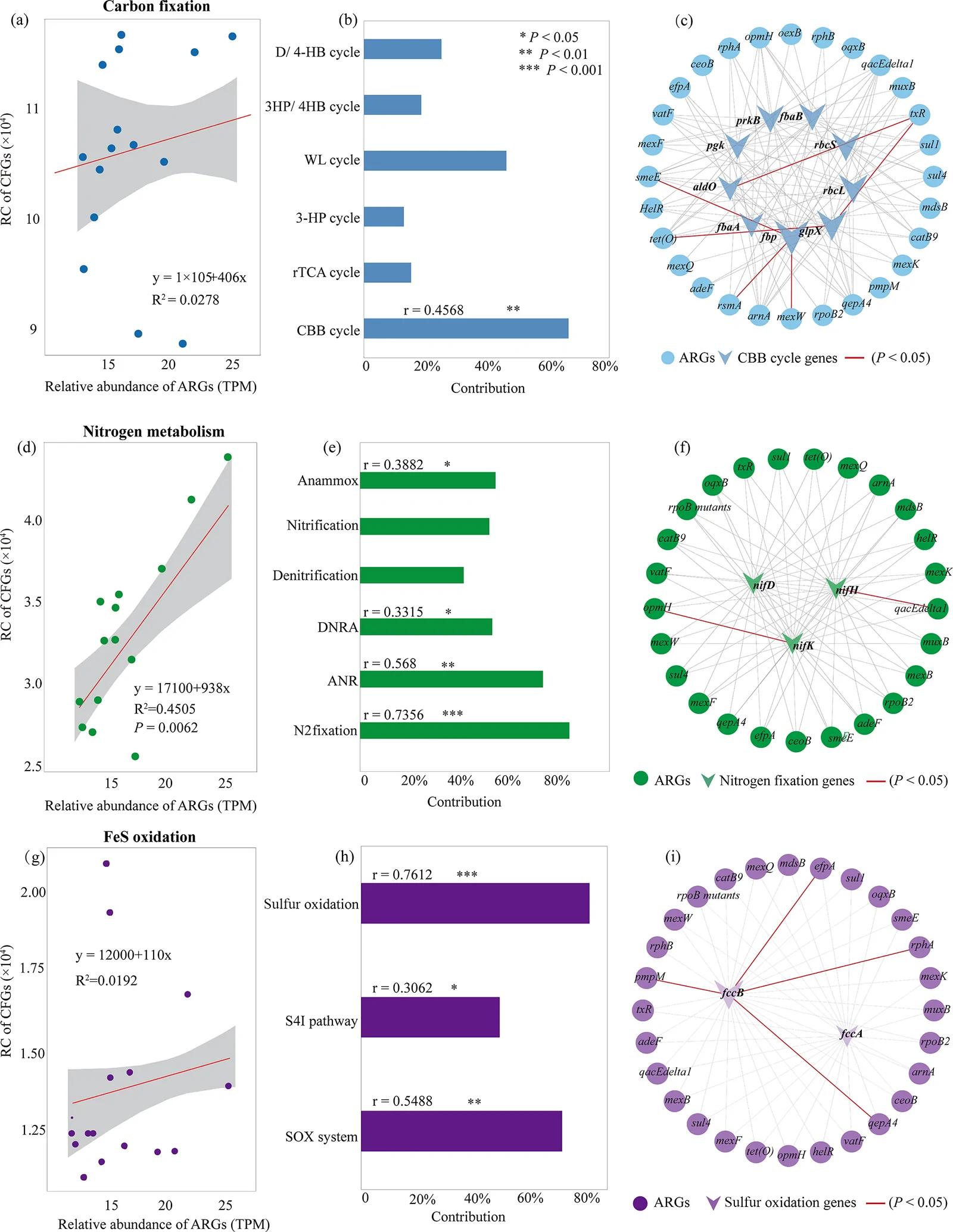
Associations between carbon fixation, nitrogen metabolism, FeS oxidation, and ARGs. (**a and b**) The association between the total abundance of ARGs and carbon fixation genes, as well as the contribution of different carbon fixation pathways to the variation in ARG abundance. The *r* and *P* values above the bars indicate correlation strength and statistical significance. (**c**) Correlation analysis between CBB cycle-related genes and ARGs. The red lines indicate significant correlations between them (*r* > 0.7, *P* < 0.05). (**d and e**) The association between the total abundance of ARGs and nitrogen metabolism genes, as well as the contribution of different nitrogen metabolism pathways to the variation in ARG abundance. The *r* and *P* values above the bars indicate correlation strength and statistical significance. (**f**) Correlation analysis between nitrogen fixation-related genes and ARGs. The red lines indicate significant correlations between them (*r* > 0.7, *P* < 0.05). (**g and h**) The association between the total abundance of ARGs and FeS oxidation genes, as well as the contribution of different FeS oxidation pathways to the variation in ARG abundance. The *r* and *P* values above the bars indicate correlation strength and statistical significance. (**i**) Correlation analysis between sulfur oxidation-related genes and ARGs. The red lines indicate significant correlations between them (*r* > 0.7, *P* < 0.05).

**Fig 5:**
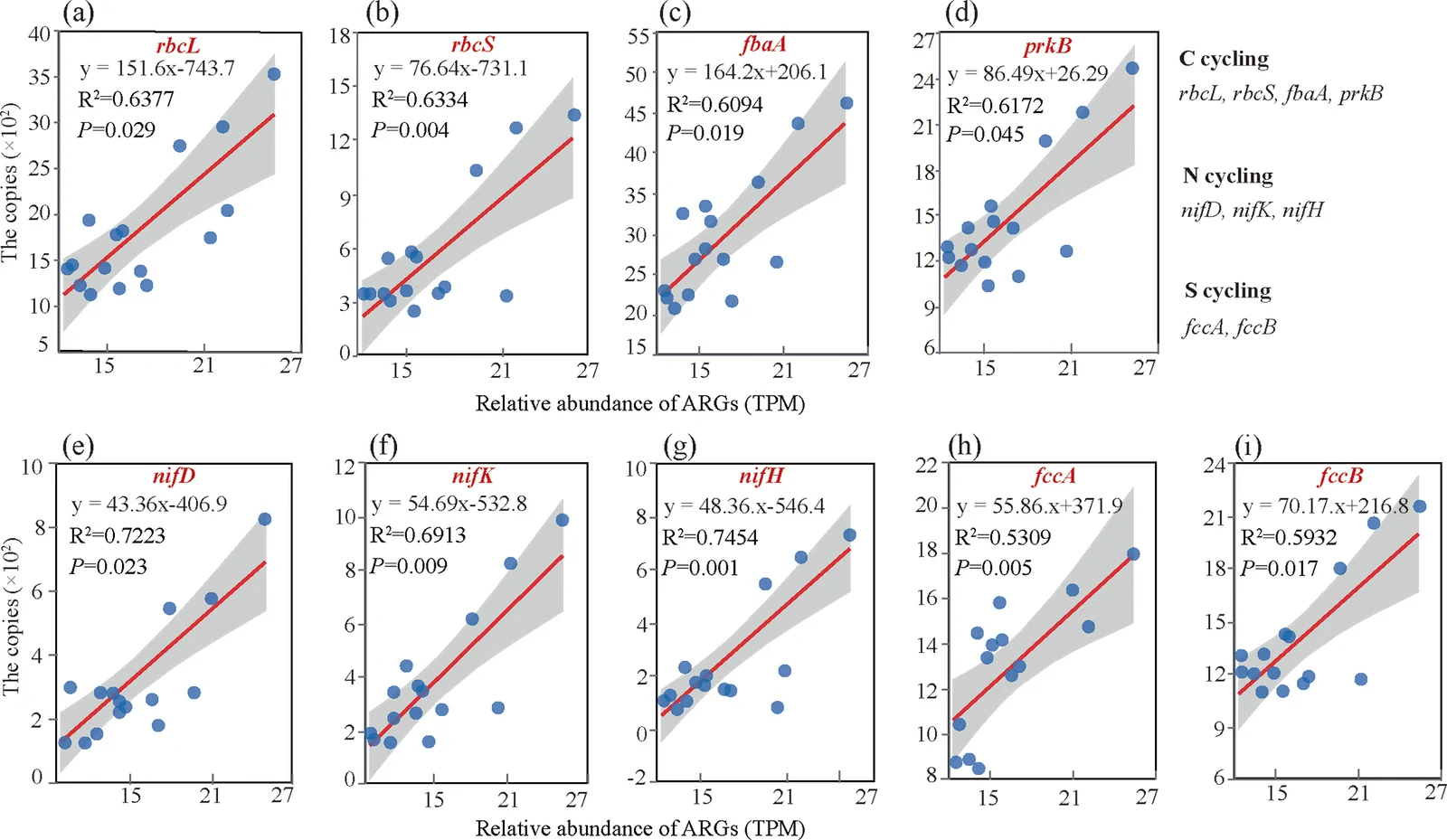
Correlation analysis of key genes in CBB cycle, nitrogen fixation, and sulfur oxidation processes with ARGs. (**a–d**) Linear regression relationship between key CBB cycle genes (*rbcS*, *rbcL*, *prkB*, and *fbaA*) and ARG abundance. (**e–g**) Linear regression relationship between key nitrogen fixation genes (*nifD*, *nifK*, and *nifH*) and ARG abundance. (**h and i**) Linear regression relationship between key sulfur oxidation genes (*fccA* and *fccB*) and ARG abundance.

CBB cycle exhibited significant positive correlations with ARGs (Fig. 4a and b). Specifically, key genes of the CBB cycle, including the aldolase gene *aldo*, the fructose-1,6-bisphosphatase gene *fbp*, and the glyceraldehyde-3-phosphate dehydrogenase gene *glpX*, showed significantly positive correlations with a subset of ARGs (Fig. 4c). Nitrogen metabolism showed a significant positive correlation with ARGs (Fig. 4d). Anammox, DNRA, ANR, and N_2_ fixation were significantly associated with ARGs, with N_2_ fixation making the highest contribution (Fig. 4e). Nitrogen fixation genes *nifK* and *nifH* were significantly associated with antibiotic resistance genes *opmH* and *qacEdelta1*, respectively (Fig. 4f). Although FeS oxidation showed no significant correlation with ARGs, thiosulfate oxidation (S4I) pathway, the SOX system, and the sulfur oxidation metabolic pathway exhibited significant positive correlations with ARGs (Fig. 4g and h). The *fccB* for sulfur oxidation was significantly associated with multiple ARGs (*pupM*, *rphA*, *efpA*, and *qepA4*; Fig. 4i). To examine how the key metabolic pathways mediated ARGs, we further analyzed the relationships between key functional genes and ARGs (Fig. 5; Fig. S4). For the CBB cycle, the key enzyme genes *rbcL* and *rbcS* for the carboxylation process, as well as the key genes *prkB* and *fbaA* for the reduction phase, exhibited significant positive correlations with ARGs (Fig. 5a through d; Fig. S4a and b). Within the nitrogen fixation pathway, *nifK*, *nifH*, and *nifD* showed significant correlations with ARGs (Fig. 5e through g). In the sulfur oxidation pathway, the flavocytochrome c sulfide dehydrogenase genes *fccA* and *fccB* showed significant positive correlations with ARGs (Fig. 5h and i).

### Sediment factors mediate ARGs

**Fig 6:**
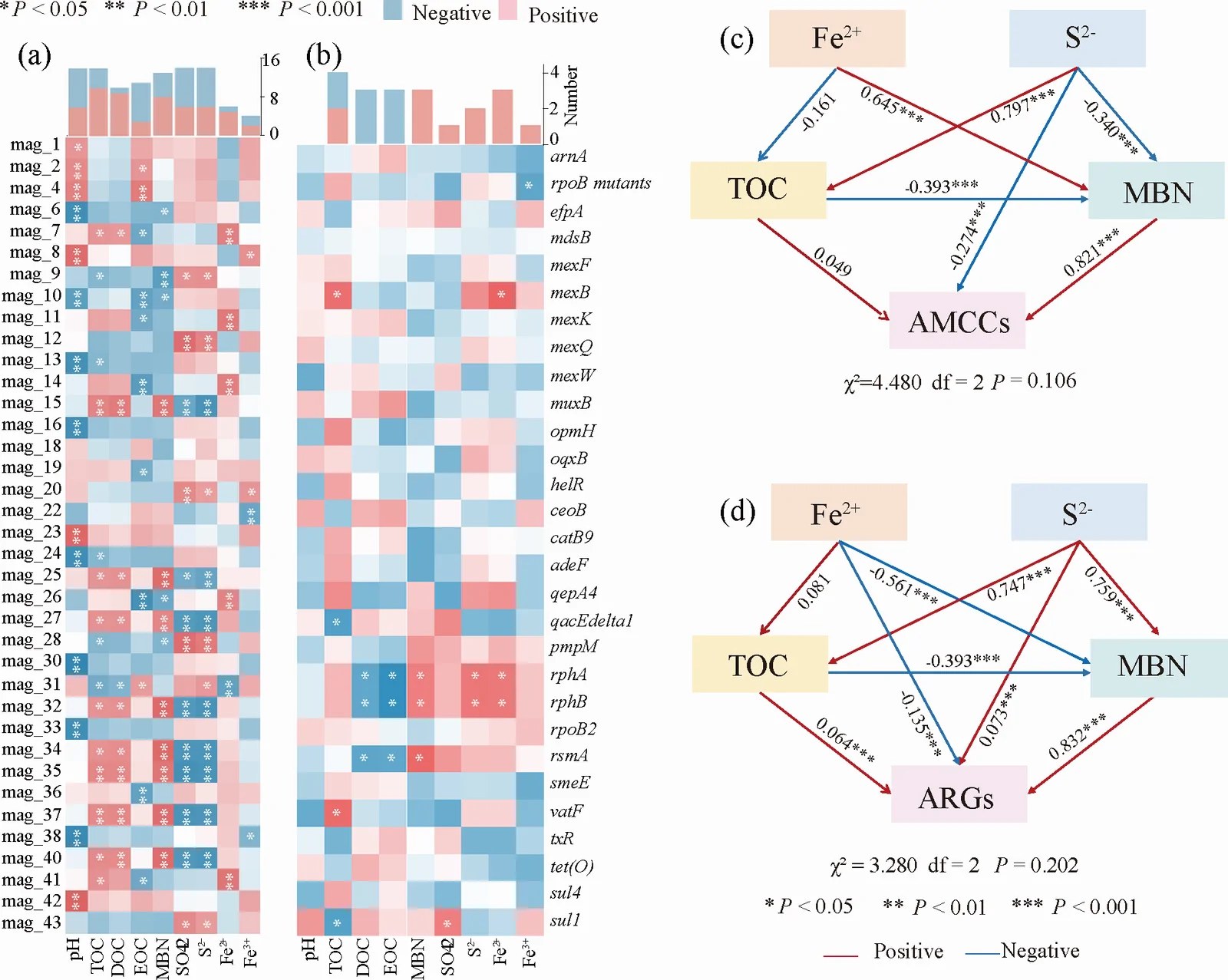
The impacts of sediment factors on ARGs. (**a**) Heatmap showing the correlation between environmental factors and metagenome-assembled genomes (MAGs) that simultaneously carry ARGs and mobile genetic elements (MGEs; *P < 0.05, **P < 0.01, and ***P < 0.001). The bar chart shows the number of MAGs significantly correlated with environmental factors, where red represents positive correlation, and blue represents negative correlation. (**b**) Heatmap showing the correlation between environmental factors and ARGs (*P < 0.05, **P < 0.01, and ***P < 0.001). The bar chart shows the number of ARGs significantly correlated with environmental factors, where red represents positive correlation, and blue represents negative correlation. (**c**) Analyze the direct and indirect relationships between S²⁻, Fe²^+^, TOC, MBN, and the abundance of MAGs carrying ARGs and MGEs based on structural equation modeling. The numerical values next to the arrows are standardized path coefficients, and *R*² represents the variance explained rate of the variables. Significance levels of regression weights were calculated using two-sided *t*-tests (*P < 0.05, **P < 0.01, and ***P < 0.001). Red represents a positive correlation, and blue represents a negative correlation. (**d**) Analyze the direct and indirect relationships between S²⁻, Fe²^+^, TOC, MBN, and the abundance of ARGs. The numerical values next to the arrows are standardized path coefficients, and *R*² represents the variance explained rate of the variables. Significance levels of regression weights were calculated using two-sided *t*-tests (*P < 0.05, **P < 0.01, and ***P < 0.001). Red represents a positive correlation, and blue represents a negative correlation.

FeS addition significantly increased concentrations of TOC, MBN, SO_4_^2–^, and Fe^2+^, but decreased EOC concentration under aerobic conditions (Fig. S5). Specific associations were observed between sediment factors and antibiotic ARGs. MBN showed significant positive correlations with multidrug and sulfonamide-resistance genes, while SO_4_^2–^ was significantly correlated with multidrug-resistance genes. Fe^3+^ was significantly associated with β-lactam resistance genes, and Fe^2+^ was significantly linked to sulfonamide resistance genes. TOC exhibited a significant correlation with peptide resistance genes. pH showed no significant correlation with ARGs, but it was associated with AMCCs (Fig. 6a and b). TOC, DOC, and EOC were significantly correlated with ARGs and AMCCs. MBN was significantly correlated with ARGs and AMCCs. SO_4_^2–^, S^2–^, Fe^2+^, and Fe^3+^ also showed positive and negative correlations with ARGs and AMCCs (Fig. 6a and b). S^2–^ not only had a significant direct positive effect on ARGs and AMCCs but can also indirectly affect the abundance of ARGs and AMCCs by regulating TOC and MBN (Fig. 6c and d).

## DISCUSSION

The “One Health” concept emphasizes that antibiotics have pressured challenge to global ecosystems and human health (41–43). With this in mind, ARGs, as emerging environmental pollutants (1, 44), have potential health risks but should not be ignored mainly due to their widespread transmission and dissemination in natural environments (2, 45). Estuarine and coastal wetlands are critical linkages between terrestrial and marine environments, which have suffered from substantial land-based pollutants and have posed regional ecological security and public health (7, 46). Sulfate respiration can yield FeS, which is generally formed in flooding and strongly reducing environments (11, 46). More importantly, redox dynamics in estuarine and coastal environments are highly coupled with sulfur and iron cycling, thereby playing a critical role in the migration and transformation of heavy metals and antibiotics (26, 46).

### FeS oxidation facilitates ARGs enrichment

The positive effects of FeS on ARGs were oxygen-dependent because FeS oxidation occurs strongly under oxygen-enriched conditions. In this study, we found that FeS significantly increased the abundance of ARGs and AMCCs under aerobic conditions. However, anaerobic conditions did not increase the abundances of ARGs and AMCCs in the presence of FeS (*P* > 0.05), which is mainly due to the limited FeS oxidation. These results indicated that FeS oxidation is significant in triggering ARG enrichment. Previous studies also reported that redox fluctuations are critical factors affecting the spatial distribution of ARGs in estuarine and coastal sediments (47). It is well acknowledged that FeS can generate ROS under aerobic conditions (13). ROS can induce oxidative stress responses in microorganisms, further increasing the recombination frequency of MGEs and promoting the horizontal transfer of ARGs (13, 48). In contrast, FeS is relatively stable and cannot trigger the aforementioned chain reaction under anaerobic conditions, which could be due to limited biogeochemical cycling of iron and sulfide. These results confirm that significantly enhanced ARG abundance was observed in oxic sediments, further emphasizing that redox fluctuation is a critical factor affecting ARGs enrichment and distribution in estuarine and coastal environments.

However, some studies have reported that chemical oxidation processes can oxidatively degrade DNA of microbial communities and further inhibit ARGs spread in aquatic environments (49). This suggests that the oxidation process of FeS under aerobic conditions may cause a tradeoff effect between enrichment and degradation of ARGs. FeS oxidation under aerobic conditions can stimulate recombination of MGEs and biogeochemical cycling, further facilitating enrichment and dissemination of ARGs (13, 16). In contrast, the oxic FeS oxidation process can degrade extracellular or intracellular DNA via Fenton-like reactions, which strongly reduces ARGs enrichments (50, 51). The contradictory results underline an environmentally dependent dynamic tradeoff mechanism of ARGs enrichments, whereby the redox dynamics of FeS, along with the composition and activity of microbial community, collectively determine whether the net effect of ARGs mediated by FeS oxidation tends to “promote dissemination” or “drive degradation.”

### Microbial community restructure favored ARGs hosts

Microorganisms are the key hosts of ARGs and directly regulate the enrichment and dissemination potential of ARGs through changes in their community composition and abundance (17, 52). In FeS-mediated sediments, the presence of FeS under aerobic conditions significantly reduced the α-diversity of microbial community but specifically increased the relative abundance of Proteobacteria. Therefore, the decreased microbial diversity would cause the enrichment of dominant taxa, which could be due mainly to the convergent community succession under environmental pressures (53). It has been well acknowledged that Proteobacteria are widely considered the main hosts and transmission vectors of ARGs in the bacterial kingdom (48). The enrichment of Proteobacteria in the presence of FeS oxidation may benefit from their strong antioxidant capacity, metal tolerance, as well as diverse energy metabolism pathways (54). In this study, host-tracking analysis strongly confirmed that the detected ARGs were mainly hosted by Proteobacteria, indicating that this phylum plays a critical role as reservoirs and transmission vectors of ARGs in FeS-enriched and aerobic environments.

Co-occurrence network analysis showed a shift in microbial interaction patterns that may affect enrichment and dissemination of ARGs. Although the complexity of the network in the presence of FeS under aerobic conditions decreased, Proteobacteria were mostly responsible for nodes (26/55) and exhibited the highest proportion of positive correlations (61%). This “simplified but more synergistic” network structure may facilitate efficient resource allocation and functional coordination, further enhancing the competitive advantage of Proteobacteria under extraneous pressures. In addition, it has been demonstrated that increasing the proportion of positive correlations between microbial networks can suggest niche overlap and functional complementarity (55), which are accompanied by metabolic coupling within Proteobacteria and between this phylum and other taxa in carbon, nitrogen, sulfur, and iron metabolisms.

### Element metabolism-driven ARGs enrichment

Biogeochemical cycles of carbon, nitrogen, sulfur, and iron through element-cycling metabolic genes are expected to have potential effects on ARGs enrichment (56, 57). The enhanced metabolic processes not only provide energy and material foundations for microbial growth but also maintain the elemental balance of ecosystems (58, 59). It is worth noting that perturbations in the element metabolism processes are accompanied by changes in the abundance and composition of ARGs (10, 60). Sulfur-mediated biogeochemical processes can significantly couple with nitrogen reduction, Fe(II) oxidation, as well as carbon fixation (61). In addition, Fe(II) can enhance carbon fixation by chemoautotrophic processes (26) and also stimulate nitrogen reduction through electron transfer (24), playing a mediating role in the coupling of carbon and nitrogen cycles. Therefore, metabolic processes of carbon, nitrogen, iron, and sulfur can substantially stimulate the host pathways of ARGs in estuarine and coastal environments, representing mechanistic linkages between elemental metabolic activities and dissemination of antibiotic resistance.

However, the influences of elemental cycles on ARGs are not independent from each other; they are coupled through multi-element collaborative mechanisms (54, 62). In this study, Proteobacteria-affiliated MAGs were enriched under aerobic conditions, in which FeS had enhanced the complete metabolic pathways for carbon and nitrogen fixation and FeS oxidation. The abundance of key genes in these three metabolic pathways was positively correlated with ARG abundance and was more significant after FeS addition. N_2_ fixation genes (*nifK*, *nifH*, and *nifD*) showed the greatest upregulation (up to 606%), and sulfur oxidation genes (*fccA* and *fccB*) and CBB cycle genes (*rbcL*, *rbcS*, *fbaA*, and *prkB*) were also significantly increased. From the perspective of energy metabolism, sulfur oxidation, as an energy-yielding process, can provide ATP and reducing power for the energy-consuming CBB cycle and N_2_ fixation (13), further creating metabolic coupling within Proteobacteria. These metabolic couplings not only enhance the adaptive advantage of Proteobacteria in FeS oxidation environments but also indirectly promote co-selection and enrichment of ARGs by increasing cellular metabolic activity and biomass turnover (60). In addition, S^2–^ not only directly and positively regulated ARGs and AMCCs but also indirectly influenced them by affecting TOC and MBN for microbial activity. These results suggest that the sulfur cycle plays a vital role in FeS-driven ARGs enrichment, creating a multi-element synergistic driving network by coupling carbon and nitrogen metabolisms.

### Environmental implications: from laboratory to field scales

In this study, we found that FeS oxidation altered microbial structure and elemental metabolic pathways, further leading to enrichment and dissemination of the ARGs. FeS addition under aerobic conditions was observed to favor the proliferation of Proteobacteria (with a 12% increase in relative abundance; Fig. S6). The positive impacts of Proteobacteria on ARGs were mainly related to the changes in three types of metabolic functions, namely sulfur oxidation, nitrogen fixation, and the CBB cycle. The abundance of key genes in the sulfur oxidation, nitrogen fixation, and CBB cycle pathways increased by 67%–606%, collectively creating the key molecular basis for regulating the enrichment and transfer of ARGs. In addition, sulfur oxidation can provide ATP and reducing power for the CBB cycle and biological nitrogen fixation, highlighting metabolic coupling within Proteobacteria. The results revealed that the total abundances of ARGs and AMCCs increased significantly after the addition of 7 mg/g FeS, whereas no obvious variation was observed in the total abundance of MGEs. This indicates that FeS enhances the horizontal transfer risk of ARGs not by increasing the abundance of MGEs but by strengthening the linkage between ARGs and MGEs on the genome.

**Fig 7:**
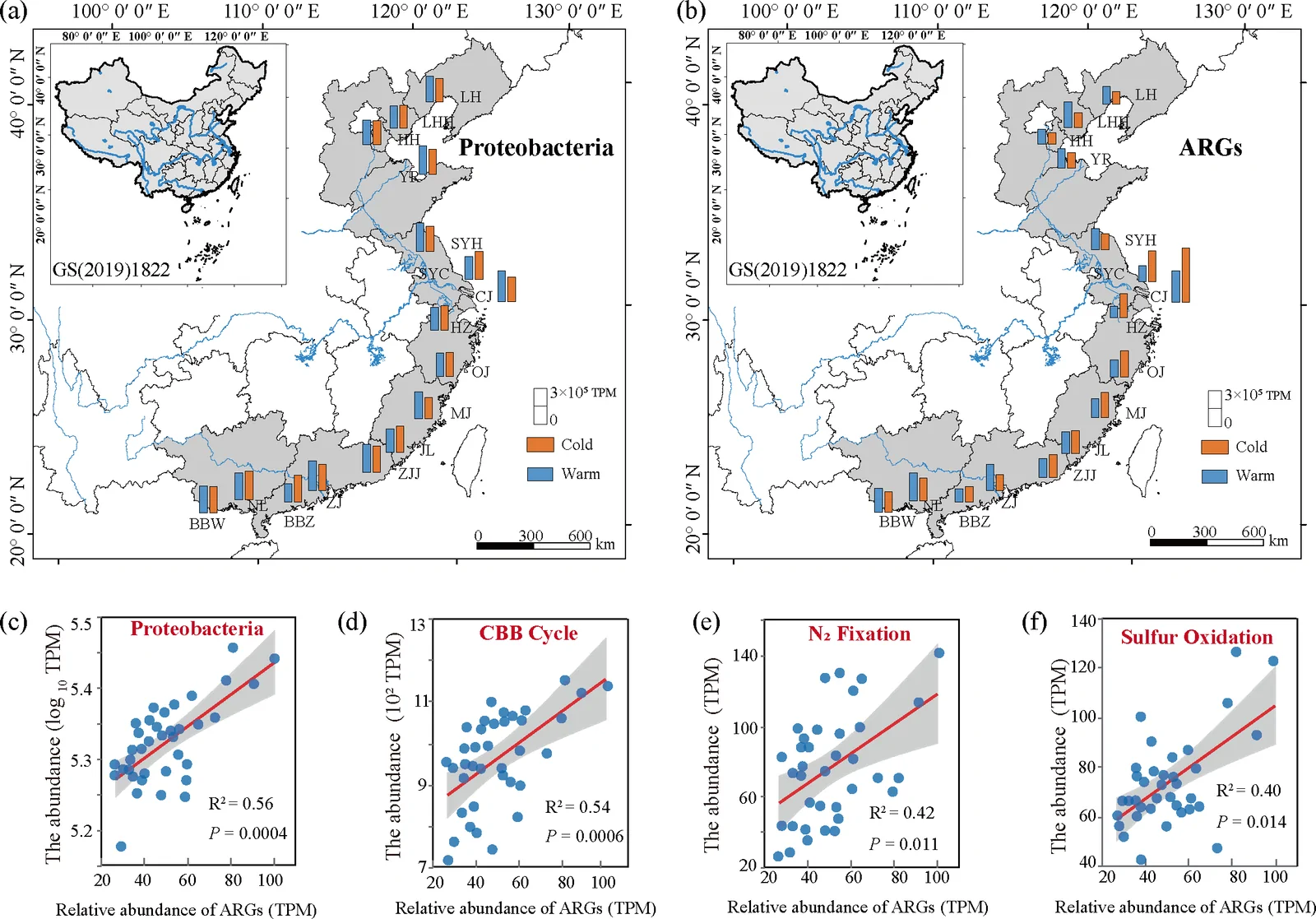
Comprehensive mechanistic elucidation of ARGs enrichment driven by FeS. (**a**) Map showing the distribution of 16 sampling sites in the estuarine-coastal zone. Bar charts reflect the relative abundance of Proteobacteria at each site in the warm and cold seasons. (**b**) Map showing the distribution of 16 sampling sites in the estuarine-coastal zone. Bar charts reflect the relative abundance of ARGs at each site in the warm and cold seasons. (**c–f**) Dot-line plots showing the linear regression between the relative abundance of ARGs and that of Proteobacteria, CBB cycle, N₂ fixation, and sulfur oxidation, respectively.

To verify the environmental generalizability of our findings, we further analyzed data collected from 16 field sites in Chinese estuarine and coastal wetlands during the cold season (March–April) and warm season (August–September) of 2019 (Fig. 7a and b). The study area was divided into southern and northern regions by the Yangtze River (CJ). The abundances of ARGs and nitrogen fixation genes were significantly higher in the southern region, while no significant differences in the abundances of carbon fixation and sulfur oxidation genes were observed between regions or seasons (Fig. S7). Results of Mantel analysis and random forest analysis showed that sediment physicochemical variables collectively explained 37.49% of the variation in ARGs, and nitrogen-related variables, including NH_4_^+^, NO_3_⁻, and NO_2_⁻, were the most important predictors for ARG variation (Fig. S8). In addition, ARG abundance was observed to significantly correlate with the abundance of Proteobacteria (Fig. 7c), suggesting the exact mechanistic linkages of FeS mediation, Proteobacteria enrichment, metabolic coupling, and ARGs increase in natural environments. It is worth noting that not all sites exhibited a synchronous increase in both variables, which may be attributed to other factors such as local sediment FeS, organic matter, and microbial structure, suggesting that this mechanism was co-regulated by multiple factors in estuarine and coastal environments. Most importantly, the CBB cycle, nitrogen fixation, and sulfur oxidation pathways were significantly correlated with the abundance of ARGs (Fig. 7d through f), further highlighting that Proteobacteria can drive the enrichment and dissemination of ARGs through multi-element metabolic synergies. Therefore, the coupling metabolic pathways have broader implications for ARGs dissemination in global estuarine and coastal environments.

### Conclusion

In this study, FeS oxidation was observed to increase enrichment and dissemination of the ARGs in estuarine and coastal sediments. The FeS oxidation reshaped microbial community structure and selectively enriched the critical Proteobacteria, which played the crucial hosts of ARGs. The enrichment and dissemination of ARGs in the presence of FeS oxidation were due to the enhanced metabolic coupling of sulfur oxidation, nitrogen fixation, and carbon fixation. CBB cycle, N_2_ fixation, and sulfur oxidation pathways were also significantly correlated with ARG abundance along the latitudinal gradient. These results highlight that FeS oxidation facilitated biogeochemical cycling and further exacerbated the enrichment and dissemination of ARGs. In addition, enrichments and disseminations of ARGs will be enhanced in the contexts of redox fluctuations, which potentially increase antibiotics risk in estuarine and coastal wetlands. The enrichment and dissemination of ARGs enhanced by biogeochemical cycling have great implications for antibiotic management in other relevant and global ecosystems. The findings and results of this study remain uncertain and limited. The microcosm incubation experiments are required to examine the effects of FeS concentration gradients, temperature conditions, and tidal cycle. In addition, the field-scale observations provide correlative evidence only, and *in situ* experiments are required to further validate the underlying mechanisms.

## Data Availability

The metagenomic sequencing reads generated in this study were deposited in the NCBI Sequence Read Archive (SRA) under accession numbers PRJNA1198723 and SRP341954.

## SUPPLEMENTAL MATERIAL

Supplemental material aem.01525-26-s0001.docx Table S1; Fig. S1 to S8.

ASM does not own the copyrights to Supplemental Material that may be linked to, or accessed through, an article. The authors have granted ASM a non-exclusive, world-wide license to publish the Supplemental Material files. Please contact the corresponding author directly for reuse.
