# Supplementary material for: Double-edged sword of FeS oxidation in estuarine and coastal sediments: accelerating elemental cycling yet enhancing antibiotic resistance enrichment
Source: Appl Environ Microbiol. 2026 Sep 30;92(10):e01525-26. doi: 10.1128/aem.01525-26 (PMC13689488; doi:10.1128/aem.01525-26)
Supplement: Supplementary file 1 [file aem.01525-26-s0001.docx]

**Supporting Information for Publication**

**Double-edged sword of FeS oxidation in estuarine and coastal sediments: Accelerating elemental cycling yet enhancing antibiotic resistance enrichment**

Wei Qiu^1,†^, Xiufeng Tang^1,†^, Boshuang Wu^2^, Haoran Huang^1^, Xiangyang Zhao^1^, Jun Yuan^1,*^, Xiaofei Li^2,*^

^1^ Jiangsu Provincial Key Lab for Organic Solid Waste Utilization, Jiangsu Collaborative Innovation Center for Solid Organic Wastes, Educational Ministry Engineering Center of Resource-Saving Fertilizers, Nanjing Agricultural University, Nanjing 210095, China,

^2^ State Key Laboratory of Estuarine and Coastal Research, East China Normal University, Shanghai 200241, China.

^*^**Corresponding author**: junyuan@njau.edu.cn (J. Yuan); xfli@sklec.ecnu.edu.cn (X. Li)

^†^Wei Qiu and Xiufeng Tang contributed equally to this work.

**Table S1**. Geochemical properties in incubated sediments of the low tidal flat

| pH | Water content （%） | SO_4_^2-^  (mg g^-1^ ) | S^2-^  (μg g^-1^ ) | Fe^2+^  (mg g^-1^ ) | Fe^3+^  (mg g^-1^ ) |
| --- | --- | --- | --- | --- | --- |
| 8.12±0.01 | 25 ±2.2 | 0.85 ±0.05 | 0.13± 0.04 | 1.52± 0.42 | 2.51± 0.06 |
| NH_4_^+^  (μg N g^-1^ ) | NO_2_^–^  (μg N g^-1^ ) | NO_3_^–^  (μg N g^-1^ ) | TOC  (mg g^-1^ ) | EOC  (mg g^-1^ ) | DOC  (mg g^-1^ ) |
| 2.15 ±0.50 | 0.19 ±0.06 | 0.76 ±0.04 | 4.67±0.01 | 3.81±0.11 | 0.04±0.02 |
| MBC  (μg g^-1^ ) | MBN  (μg g^-1^ ) | Clay (%) | Silt (%) | Sand (%) | Density  (g cm^-3^ ) |
| 113.00±7.80 | 2.46±0.13 | 1.00±0.12 | 85.00±6.10 | 14.00±1.60 | 1.54 ±0.12 |

**Note**: TOC, total organic carbon; EOC, easily oxidizable carbon; DOC, dissolved organic carbon; MBC, microbial biomass carbon; MBN, microbial biomass nitrogen.

**Figure S1**. Study area and sampling site in Yangzte Estuary.

**Figure S2.** Experimental design and flowchart. A total of 300 g of sediment samples were incubated in the dark at 25 °C for a 91-day long-term experiment, with deionized water replenished as needed in the aerobic treatments to maintain approximately 40% water content. Five treatments were established: control group (CK), aerobic group (Aerobic), anaerobic group (Anaerobic), aerobic group amended with iron sulfide (Aerobic-FeS), and anaerobic group amended with iron sulfide (Anaerobic-FeS). Environmental factors, amplicon sequencing, and metagenomic sequencing data were determined at the end of incubation. In addition, 16 representative sampling sites were selected across the estuarine and coastal zone, where sediment samples were collected in warming and cold seasons for metagenomic sequencing analysis.

**Figure S3.** Effects of FeS on bacterial community. (a) Non‑metric multidimensional scaling (NMDS) analysis was performed to examine the effects of FeS treatment on microbial community structure under aerobic and anaerobic conditions. (b) Alpha‑diversity indices (ACE, Chao1, Shannon, Simpson) of bacterial communities under different treatments. All indices were calculated based on the normalized ASV table. In the box plots, the horizontal line represents the median, whiskers indicate the normal data range (typically extending to 1.5 times the interquartile range), and circles denote outliers. Different letters indicate significant differences between treatments based on the Tukey–Kramer HSD test following ANOVA (*P* < 0.05). (c) Source tracking of antibiotic resistance genes using a Sankey diagram (left: ARGs; right: microbial sources at the phylum level).

**Figure S4** Functional gene profiles of CBB cycle, nitrogen fixation, sulfur oxidation, and their correlation with ARGs. (a) Schematic diagram of the CBB cycle, nitrogen fixation and surfur oxidation metabolic pathways and its corresponding key genes for each step. (b) Linear regression relationship between key CBB cycle genes (*fbp*, *fbaB*, *pgK*, *glpX*, *aldO*) and ARGs abundance.

**Figure S5.** Effects of FeS addition on environmental factors and ARGs under aerobic and anaerobic conditions. (a-i) The effect of adding FeS under aerobic and anaerobic conditions on nine environmental factors (pH, TOC, DOC, EOC, MBN, SO₄²⁻, S²⁻, Fe²⁺, Fe³⁺) concentrations. In the box plots, the horizontal line represents the median, the whiskers indicate the normal data range (usually 1.5 times the interquartile range), and circles denote outliers. Different letters indicate significant differences between treatments based on Tukey-Kramer HSD test after variance analysis (P < 0.05). (j) Correlation network between nine environmental factors (pH, TOC, DOC, EOC, MBN, SO₄²⁻, S²⁻, Fe²⁺, Fe³⁺) and nine categories of antibiotic resistance genes (tetracyclines, fluoroquinolones, MLS, multidrug-resistant, peptides, phenols, rifamycins, sulfonamides, and triclosan). Blue nodes represent environmental factors, pink nodes represent antibiotic categories, and red lines indicate significant correlations between them (r > 0.7, *P* < 0.05).

**Figure S6.** Schematic diagram illustrating how FeS promotes ARGs enrichment in an aerobic environment by driving the enrichment of Proteobacteria and synergizing three metabolic pathways: sulfur oxidation, nitrogen fixation, and the CBB cycle. Red arrows represent an upward trend.

**Figure S7.** Sampling sites in the estuarine and coastal region were divided into southern and northern groups by the Yangtze River, including seven sampling sites in the southern region and five sampling sites in the northern region. We compared the abundance patterns of ARGs, sulfur oxidation, N₂ fixation, and the CBB cycle between the southern and northern regions, as well as between cold and warm seasons. (* *P* < 0.05, ** *P* < 0.01, *** *P* < 0.001).

**Figure S8.** (a) Correlations between ARGs and sediment physicochemical parameters based on Pearson and Mantel correlation analyses; (b) Relative importance of predictor variables for ARGs was evaluated using a random forest model. MC: moisture content; TOC: total organic carbon; TC: total carbon; TN: total nitrogen. (* *P* < 0.05, ** *P* < 0.01, *** *P* < 0.001).
